# The Modernising of Old Buildings

**Published:** 1912-06-15

**Authors:** 


### HOSPITAL ARCHITECTURE AND CONSTRUCTION.

[Communications on this subject should be marked "Architecture" in the left-hand top corner of the envelope.]

### The Modernising of Old Buildings.

Probably there is no subject on which less is written and more is thought than the wisdom or unwisdom of adapting existing buildings. It may be that a committee is by some means convinced that premises under its control are in need of remodelling to bring them into line with modern standards, and an architect is therefore instructed to examine and report in the matter. He, on his dexter side, has most likely a high standard of what hospital and allied buildings should be, and on his sinister side the question of cost. How is he to reconcile the two standpoints?

It may be said that perfection in the design of hospital works knows no end; but on the other hand cost does, as a general rule, determine how far the architect may go. To illustrate the indefiniteness of our standards of perfection we may recall an interesting paper read before the Royal Society of Arts on February 7 by Messrs. Leonard Hill, M.B., F.R.S., and Martin Flack, M.A., M.B., B.Ch. The conventional theories with respect to what may be termed the natural philosophy of ventilation are to some extent assailed by the authors of this paper, whose conclusions, whether they be accepted or not, are based on painstaking investigation, and are therefore specially worthy of the close and earnest attention of all to whom the subject is of interest. The authors of the paper attack some accepted theories of ventilation and the relative standard of $CO_2$ legally permissible where numbers of persons are employed, arguing that a powerful deodoriser such as ozone will give the necessary impetus both to the cutaneous and respiratory systems which is desirable for health and comfort. This is one item of possible development which in its course may lower the present high standard of cubic area per patient in the ward; and there are many others.

### The Effects of Legislation.

The architect in forming an opinion on the buildings must have foremost in his mind their purpose, for it is evident that the standard for the remodelled workhouse infirmary, where most of the patients may be chronic cases, will be entirely different from that of an infectious hospital. In London it sometimes happens that alterations are made imperative owing to legislation providing for alternative means of escape in case of fire, and this sometimes introduces larger questions of remodelling; but the modern ward should have, apart from legislation, means of escape at two opposite points from each floor. It frequently happens also that the sanitary blocks are placed on the staircase landings half-way between floors, and there is no provision for cut-off ventilation. This is undesirable, as patients have to go up or down to them, and the want of a cut-off ventilating bridge means that a contaminating influence to the atmosphere must exist. One of the most usual defects in an old building is the ward floor; open joints are numerous, and if originally constructed with iron tongues the upper edge of the floor boards breaks away, developing germ-accumulating interstices; of all defects this is probably the least objectionable; for the composite floors now on the market—and their name is legion—work best of all when laid on a very rough surface.

### The Window Question.

A serious fault, judging from modern standards, is the small window-area usually found in old buildings. To enlarge the openings is a costly item, but a good compromise can sometimes be effected by inserting new window-framings which take up less area than the original woodwork, and to lower the sills, leaving the existing jambs and lintels. Sometimes wards are found without any plaster on the walls, and the brickwork is either painted or distempered; this, however, can be easily remedied by raking out the joints to provide a key for new plasterwork; the only detail requiring careful consideration is the finish of plasterwork against the window-frames.

A common virtue in old buildings is that they are generally heated and ventilated on the open-fire system; there is none better, and it is only necessary in modernising to insert a new slow-combustion fireplace, simple and comely in design and radiating a greater heat than the old-fashioned cast-iron interiors are capable of.

### Adapting Double Wards.

One of the most difficult problems for the architect is to decide what to do with the double wards. They are unsatisfactory from every standpoint, and the spinal division is probably their worst feature; without gutting the whole block and putting in new floors, which are very costly owing to the large span, it is almost impossible to do away with the supporting piers, and these invariably contain some of the fireplace flues. In such a case the architect will probably do well to consider the possibility of adapting these old wards to administrative purposes, and to build new ward pavilions on adjoining portions of the site. But here again is a new factor: the very size of the site may be a determining condition; if large, it may lend itself to such a scheme; if small, it may either provide an argument for the remodelling of the existing wards or may serve as an equally strong plea for abandoning any attempt at remodelling. In fact, a restricted area of land may lead the architect to the conclusion either that the site should be abandoned altogether or that the old fabric should be entirely supplanted by a new one, so as to make the fullest and best use of the ground at disposal.

Generally, perhaps, modernising answers where the problem is relatively minor. The conversion of old wards to administrative purposes is more truly an abandonment than a conversion, as a little reflection will show.

